# Gene cloning of a neutral ceramidase from the sphingolipid metabolic pathway based on transcriptome analysis of *Amorphophallus muelleri*

**DOI:** 10.1371/journal.pone.0194863

**Published:** 2018-03-28

**Authors:** Lin Zhong, Erxi Liu, Chaozhu Yang, Ying Diao, Nunung Harijati, Jiangdong Liu, Zhongli Hu, Surong Jin

**Affiliations:** 1 State Key Laboratory of Hybrid Rice, College of Life Sciences, Wuhan University, Wuhan, Hubei, PR China; 2 Institute of Konjac, Enshi Academy of Agricultural Sciences, Enshi, Hubei, PR China; 3 Lotus Engineering Research Center of Hubei Province, College of Life Sciences, Wuhan University, Wuhan, Hubei, PR China; 4 Department of Biology, Faculty of Mathematics and Natural Sciences, Brawijaya University, Jl.Veteran Malang, Indonesia; 5 College of Life Science, Department of Biology, College of Life Sciences, Wuhan University, Wuhan, Hubei, PR China; 6 Institute of Chemical and Life Science, Wuhan University of Technology, Wuhan, Hubei, PR China; University College Dublin, IRELAND

## Abstract

*Amorphophallus* is a perennial herbaceous plant species mainly distributed in the tropics or subtropics of Asia and Africa. It has been used as a traditional medicine for a long time and now is utilized for the pharmaceutical, chemical and agriculture industries as a valued economic crop. Recently, *Amorphophallus* has attracted tremendous interest because of its high ceramide content. However, the breeding and genome studies are severely limited by the arduous whole genome sequencing of *Amorphophallus*. In this study, the transcriptome data of *A*. *muelleri* was obtained by utilizing the high-throughput Illumina sequencing platform. Based on this information, the majority of the significant genes involved in the proposed sphingolipid metabolic pathway were identified. Then, the full-length neutral ceramidase cDNA was obtained with the help of its candidate transcripts, which were acquired from the transcriptome data. Furthermore, we demonstrated that this neutral ceramidase was a real ceramidase by eukaryotic expression in the yeast double knockout mutant Δypc1 Δydc1, which lacks the ceramidases—dihydroCDase (YDC1p), phytoCDase (YPC1p). In addition, the biochemical characterization of purified *A*. *muelleri* ceramidase (AmCDase) exhibited classical Michaelis-Menten kinetics with an optimal activity ranging from pH 6.5 to 8.0. Based on our knowledge, this study is the first to report the related information of the neutral ceramidase in *Amorphophallus*. All datasets can provide significant information for related studies, such as gene expression, genetic improvement and application on breeding in *Amorphophallus*.

## Introduction

*Amorphophallus* is a member of the family *Araceae*, order of *Alismatales*, and mainly distributed in the tropics or subtropics of West Africa and South Asia [1–3]. *Amorphophallus konjac* and *Amorphophallus albus* are widely grown around the world [4, 5]. The corm of *Amorphophallus* is rich in glucomannan and ceramide, but it frequently suffers from weak disease resistance and a low coefficient of propagation [6]. By contrast, *A*. *muelleri* has high disease resistance and is capable of improving the reproductive rate during relay growth, and its corm, bulbils and seeds can be used as propagative organs to shorten the growing cycle [7]. Moreover, the plant can be applied as a substitute to opium poppy plantations because of the similarity in the growing environment, which can eliminate this source of narcotics. As a result, *A*. *muelleri* is an *Amorphophallus* species with the highest competitiveness and economic value. However, the cultivation and development of *A*. *muelleri* is severely delayed by the limited studies on it. Currently, a new high-throughput sequencing technology has been developed to excavate abundant information, which could drastically enhance *Amorphophallus* studies. Sequencing-by-synthesis (SBS) is based on the Illumina sequencing platform, it has been widely used in many life science fields, including biomedicine, transcriptomics and agriculture [4].

Based on transcriptome analysis by the high-throughput sequencing technology, the sphingolipid metabolic pathway of *A*. *muelleri* could get the better study. Sphingolipids are a diverse group of lipid molecules on the structure, which can be found in various organisms of eukaryotes and bacteria [8]. Although there are numerous types of sphingolipids among different species, the basic building block is a long-chain sphingoid base (LCB). Sphingolipids are integral components of plant membranes that account for a certain proportion of plant membrane lipids [9, 10]. Basically, plant sphingolipids are usually grouped into four main categories: ceramides, glycosyl inositol phosphoceramides (GIPCs), free LCBs, and glycosylceramide plants [11]. Ceramidases degrade ceramides to sphingosine by hydrolysing the N-acyl linkage between the LCB and the fatty acid [12]. In general, ceramidases are classified into three types, including acid, neutral and alkaline ceramidases, according to the pH that is optimal for their activity [11, 13]. The metabolites of ceramide, such as sphingosine and sphingosine-1-phosphate, play important roles in various cellular processes governing growth, differentiation, stress responses and apoptosis [11, 14, 15]. However, because of inadequate preparation for the theory and a lack of thorough studies, the regulatory effects of sphingolipid metabolism in cellular processes are still poorly understood. In our study, high-throughput sequencing by Illumina Hiseq^™^ 2500 was used to gain more molecular biology data from *Amorphophallus*. The bioinformatic analysis of the *A*. *muelleri* transcriptome provided references for screening candidate genes of the possible sphingolipid metabolic pathway and cloning the full-length cDNA sequence of the key enzyme (neutral ceramidase). In addition, the preliminary biochemical characterization of the neutral ceramidase of *Amorphophallus* (AmCDase) indicated that this enzyme was a real functional ceramidase that could utilize D-erythro-C12-NBD-ceramide as a substrate.

## Materials and methods

### Material preparation

No specific permissions were required for the locations where we collected wild materials because *A*. *muelleri* is naturally distributed in Indonesia and is also widely grown for food. We confirm that the field studies did not involve endangered or protected species.

The *A*. *muelleri* materials were collected from a field in Malang, Indonesia (112°40′41″E, 8°3′31″N) in September 2015. The fresh leaves from five four-month-old plants (the size of 5.5 cm×2.5 cm) were gathered and mixed to minimize the influence of transcriptome variability among individual plants, and then immediately frozen using liquid nitrogen and stored at -70 °C.

### mRNA sequencing and bioinformatics analysis

The total RNA was extracted and kept for further analysis. The construction and sequencing of the cDNA library was performed by the Illumina Hiseq^™^ 2500 (Illumina) (San Diego, CA) at the Shanghai Hanyu Bio-Tech company, Shanghai, China (http://www.hanyubio.com) according to the manufacturer’s instructions. The results were submitted into the NCBI SRA database (SRP081028). Trinity software was used to assemble the reads from purity filtering and initial quality tests [16]. Generally, Trinity consists of three separate modules, including Inchworm, Chrysalis and Butterfly, and these modules process large-scale data sequentially. The reads are combined into longer fragments, with all the redundancy for subsequent analysis to be removed, and the longest sequences are defined as Unigenes. Finally, the Unigenes obtained by Trinity were submitted into protein databases, including GenBank NR, GO (gene ontology), KEGG (Kyoto Encyclopaedia of Genes and Genomes), and KOG (The Eukaryotic Clusters of Orthologous Groups) databases for comparison using the BLASTX algorithm (e-value ≤ 1e-5), and the best match was selected for the comment information [4, 17].

### Cloning full-length cDNA of AmCDase

The full-length cDNA of AmCDase was cloned by rapid amplification of the cDNA ends (RACE). The RACE experiment followed the protocol of the SMART RACE cDNA Amplification Kit (Clontech, Palo Alto, California, USA). Based on the assembled transcripts, Gene RACE Primers (GRPs) and Gene Special Primers (GSPs) were designed to amplify the full-length cDNA of AmCDase. Nested Primers (NGSPs) were designed in case neither the GRP nor GSP could generate a satisfactory product. In addition, the gene was re-amplified by PCR using a pair of primers (CGPs) which were designed according to the sequences of the 5’- and 3’-ends (Table 1). After the PCR products were cloned and sequenced, the online BLAST server (http://blast.ncbi.nlm.nih.gov/Blast.cgi) was used to confirm the identity of the neutral ceramidase gene. Open reading frames (ORFs) and derived amino acid sequences were estimated by the ORF Finder (http://www.ncbi.nlm.nih.gov/projects/gorf/). The alignments of the DNA and protein sequence were performed by the DNAMAN Program, and phylogenetic tree analysis was accomplished by MEGA 5.1 [18]. These full-length nucleotides and the corresponding amino acid sequences of AmCDase cDNA were submitted to GenBank.

**Table 1:** Primers for amplifying cDNA of AmCDase.

Primer name	Nucleotide sequences
GRP1	5'-TATCTGAAGTGGAAGTATAGAAG-3’
GRP2	5‘-CAGGTACACTGAGGATGACCAGC-3’
GSP1	5'-TCCGATTACCTGGTAGGGCTCGCG-3'
GSP2	5'-TCTTGGGGATCCGCCACTCGATGG-3'
NGSP1	5'-TATCTGAAGTGGAAGTATAGAAG-3’
NGSP2	5'-CAGGTACACTGAGGATGACCAGC-3’
CGP1	5'-CTTACTGGCTTATCGAAATTAATA-3’
CGP2	5'-GTGTACCTTAGCTATTTTCTGAGC-3'

### Heterologous expression in the yeast double knockout mutant

The AmCDase coding sequence was subcloned into the yeast expression vector pYES2/CT, which was regulated by the Gal1 promoter, and the construct was transformed into the double knockout mutant of yeast Δypc1 Δydc1 by the lithium acetate method [4, 19, 20]. The yeast strains containing pYES2-AmCDase were grown in synthetic basic culture medium with plenty of glucose and uracil-deficient supplementation. The yeast transformed with empty pYES2/CT vector was assigned to the control group. Then, the yeast was cultured in 2% SC-Ura^-^medium (w/v) with galactose and the cells were collected at different culture time points. To identify the best time for the induction of AmCDase, the amount of recombinant AmCDase was determined by Western blotting using anti-His antibody. The yeasts at different culture time points were solubilized in SDS/PAGE sample buffer, and the cell lysates were isolated as mentioned previously [21]. The homogenates were assayed for the following tests in which D-erythro-C12-NBD-ceramide was used as a substrate and its content was analysed by reverse-phase HPLC (High Performance Liquid Chromatography) with DAD detector (Determination conditions: Sino Chrom C18 reversed-phase column, Agilent 1100 chromatographic system, detector wavelength of 230 nm, column temperature 25 °C, acetonitrile as the mobile phase, sample size 10 μl) and the standard curve was consequently obtained. To test the biochemical properties of recombinant AmCDase, , the samples were initialized with the same amount of D-e rythro-C12-NBD-ceramide as a substrate, but the different amounts of recombinant AmCDase that reacted with the substrate were added into these samples. D-erythro-C12-NBD-ceramide was diluted at a concentration of 100 μM (3.13 mol %) in 100 mM phosphate buffer, pH 5.7, containing 0.2% Triton X-100, and 5 mM MgCl_2_ in a total volume of 100 μl. The enzymatic reaction lasted 1 h at 37 °C, and was terminated by the addition of chloroform/methanol (1:1, v/v) [9]. The enzymatic reaction was started by the addition of the same amount of cell lysate, and a remnant amount of D-erythro-C12-NBD-ceramide in different samples after the reaction was analysed under the same conditions.

### Purification and preliminary biochemical characterization of AmCDase

AmCDase genes were cloned into pET28a encoding a 6×His tag. Then, pET28a-AmCDase was transformed into *E*. *coli* strain BL21 (DE3)-pLysS cells and cultured at 37 °C in 200 ml of Luria-Bertani medium containing 100 μg/ml of Kanamycin to OD_600_ = 0.5. The expression of protein was induced by the addition of 0.1 mM isopropyl 1-thio-D-galactopyranoside. Then, the cells were harvested and suspended in 20 ml of 10 mM Tris-HCl buffer, pH 7.4, containing 0.15 M NaCl and 0.1% Triton X-100. After that, the cell debris was sonicated for 2 mins, and removed by centrifugation (8000 × g for 10 min). The supernatant was loaded onto the nickel nitrilotriacetic acid resin (Cwbiotech, cat. no. cw0010A), and the column was washed with 20 mM phosphate buffer, pH 7.4, containing 0.15 M NaCl, 0.05% Triton X-100, and 20 mM imidazole. The enzyme was eluted by 150 mM imidazole in elution buffer (20 mM sodium phosphate buffer, pH 7.4, containing 0.15 M NaCl, 0.05% Triton X-100). The eluted fractions were pooled and loaded onto a Superdex 200 HR column (GE Healthcare) equilibrated with 25 mM HEPES buffer, pH 7.5, containing 100 mM NaCl.

Subsequently, the biochemical properties of the purified AmCDase were tested. The substrate was further dissolved in various buffers at different pH values to determine the pH-dependency of AmCDase: 100 mM acetate buffer, pH 3.95–5.83; 100 mM phosphate buffer, pH 6.04–7.16; and 100 mM Tris buffer, pH 7.21–8.84. Then, the effects of various cations on AmCDases were examined after sustaining the reactions at 37°C for 1 hour. The specific activity was obtained by standard curves with known concentrations of D-erythro-C12-NBD-ceramide and all the measurements for each sample were repeated three times independently.

## Results and discussion

### Preliminary analysis of *A*. *muelleri* transcriptome

At first, a total of 31,734,628 raw sequencing reads were produced, then 30,264,334 (95.4%) reads of *A*. *muelleri* were harvested through Illumina HiSeq 2500 high-throughput sequencing with an average length of 150 bp, after the rigorous quality review and data filtering. The size of the sequencing data was approximately 4.76 G; the Q20 ratio (sequencing error rate<1%) was 97.63% and the GC proportions was 50.28% (S1 Table). Trinity efficiently reconstructed the transcriptome for the *de novo* assembly [16]. Based on the high-quality reads, a total of 85,775 transcripts were assembled with an average length of 794.77 nt. The longest transcript was selected as a unigene in every subgraph. The reads of *A*. *muelleri* were assembled into 58,858 unigenes with an average length of 618.30 nt. Transcript lengths ranged from 201 bp to 8330 bp. Transcripts with lengths between 200 to 300 nt accounted for 47.11% of the total reads, whereas transcripts between 2500 to 4000 nt only accounted for 2.48% (S1 Fig).

#### Transcript functional annotation

As is known to all, longer sequences were more conducive to bioinformatic analysis. Consequently, further analysis was mainly based on the longest sequences, which were also called all-unigenes. To validate and annotate the assembled all-unigenes, a sequence similarity search was conducted against the non-redundant (NR), EuKaryotic Orthologous Groups (KOG), Kyoto Encyclopaedia of Genes and Genomes (KEGG) and Swissprot databases using the BLASTX algorithm. The results indicated that 21,944 all-unigenes exhibited gene annotation in at least one database (S2 Table). A total of 10,385 all-unigenes can be annotated in all the databases, whereas 36,914 all-unigenes (62.72%) could not be annotated in any database. The non-annotated all-unigenes may be considered new genes.

#### Functional classification by KOG and GO

KOG is eukaryote specific version of the Clusters of Orthologous Groups database, which was used to align the all-unigenes for predicting their potential functions. Per the Nr hits, a total of 11851 sequences were specified to 25 categories in the KOG database. Clusters of “General function prediction” took the largest proportion (1386, 11.70%), followed by “Posttranslational modification, protein turnover, chaperones” (1349, 11.38%) and “Signal transduction mechanisms” (1200, 10.13%) groups. The smallest categories included the cell motility, lipid transport and metabolism (3; 0.03%), extracellular structures (6; 0.05%), and nuclear structures (7; 0.06%) (S2 Fig).

**Fig 1:**
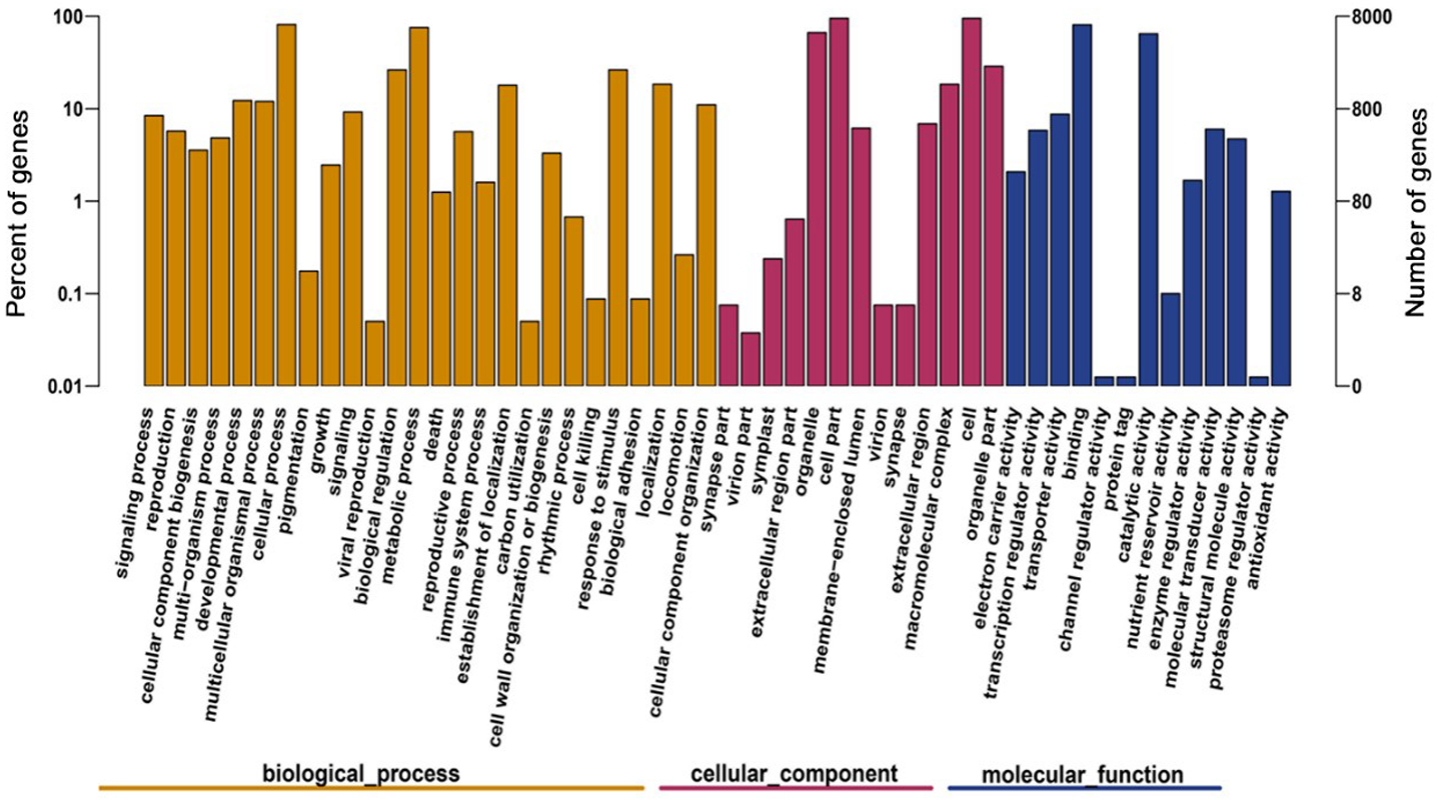
Gene function classification (GO).

GO functional annotation can be derived from the Nr annotation information. The GO classification system includes three broad categories: molecular function, biological process, and cell components. These categories were further divided into 52 small categories, and a total of 14,060 (21.39%) all-unigenes were assigned to 13 subcategories in cellular components, 25,399 (38.64%) all-unigenes were assigned to 13 subcategories in molecular function, and 26,264 (39.96%) all-unigenes were assigned to 26 subcategories in biological processes. As seen from the result, the biological process category had the largest number of genes, including signalling processes (7583), reproduction (7583), and cellular component biogenesis (6508). Only a few all-unigenes were assigned to molecular transducer activity (3), antioxidant activity (1), proteasome regulator activity (1) and structural molecule activity (1) (Fig 1).

#### Metabolic pathways analysis by KEGG

**Fig 2:**
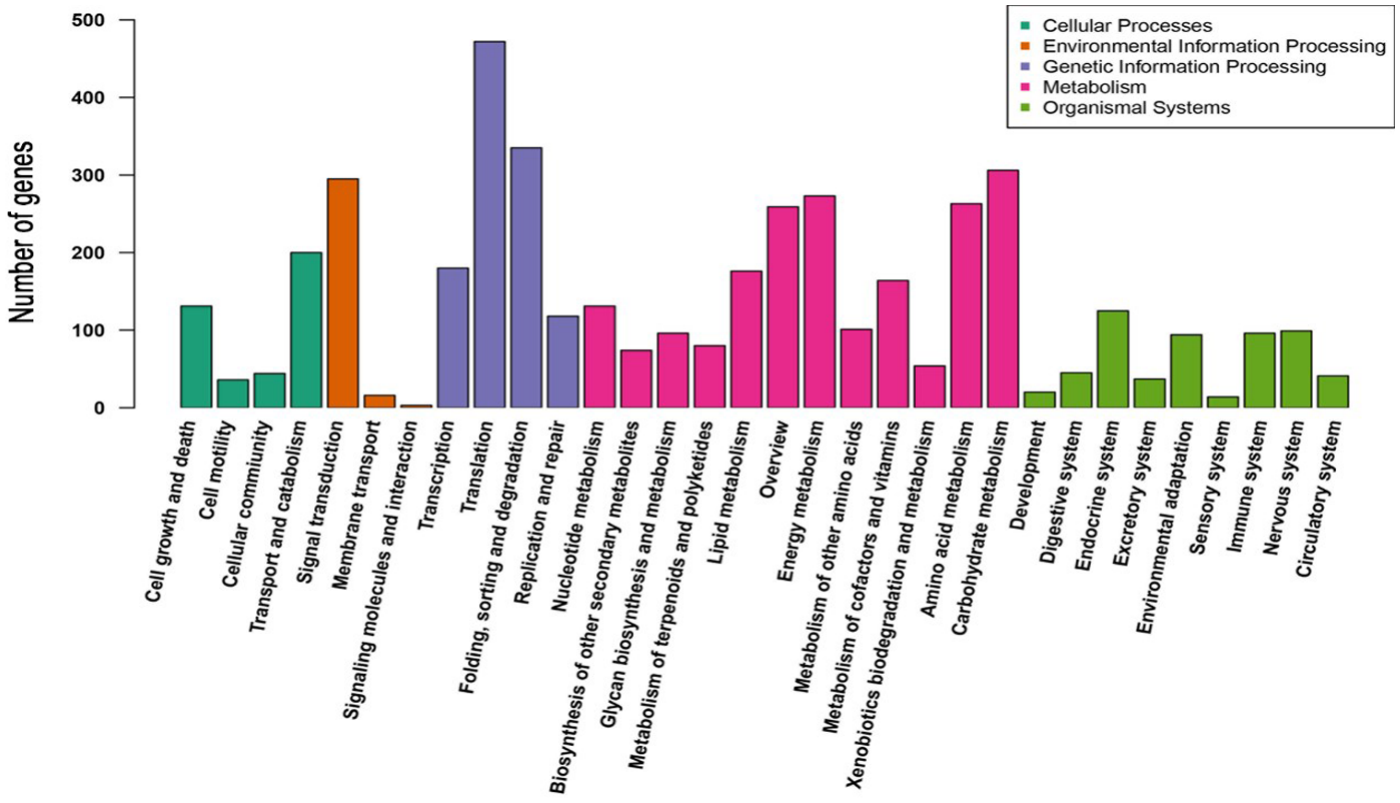
Pathway annotation and classification of contigs (KEGG).

To give a better understanding of the biological pathways in *A*. *muelleri*, 21,239 all-unigenes were mapped to 271 paths in the KEGG database. These all-unigenes were divided into five categories: cellular processes, metabolism, organismal systems, genetic information and environmental information processing. Most of the all-unigenes were assigned to “Metabolism” and “Genetic Information Processing”. As shown in Fig 2, the KEGG metabolic pathways involved 32 subcategories, such as carbohydrate metabolism, signal transduction, translation, lipid metabolism and amino acid metabolism. These results provided valuable information for investigations on the cell processes and enzyme functions of *A*. *muelleri*.

#### Construction of the possible sphingolipid metabolic pathway

**Fig 3:**
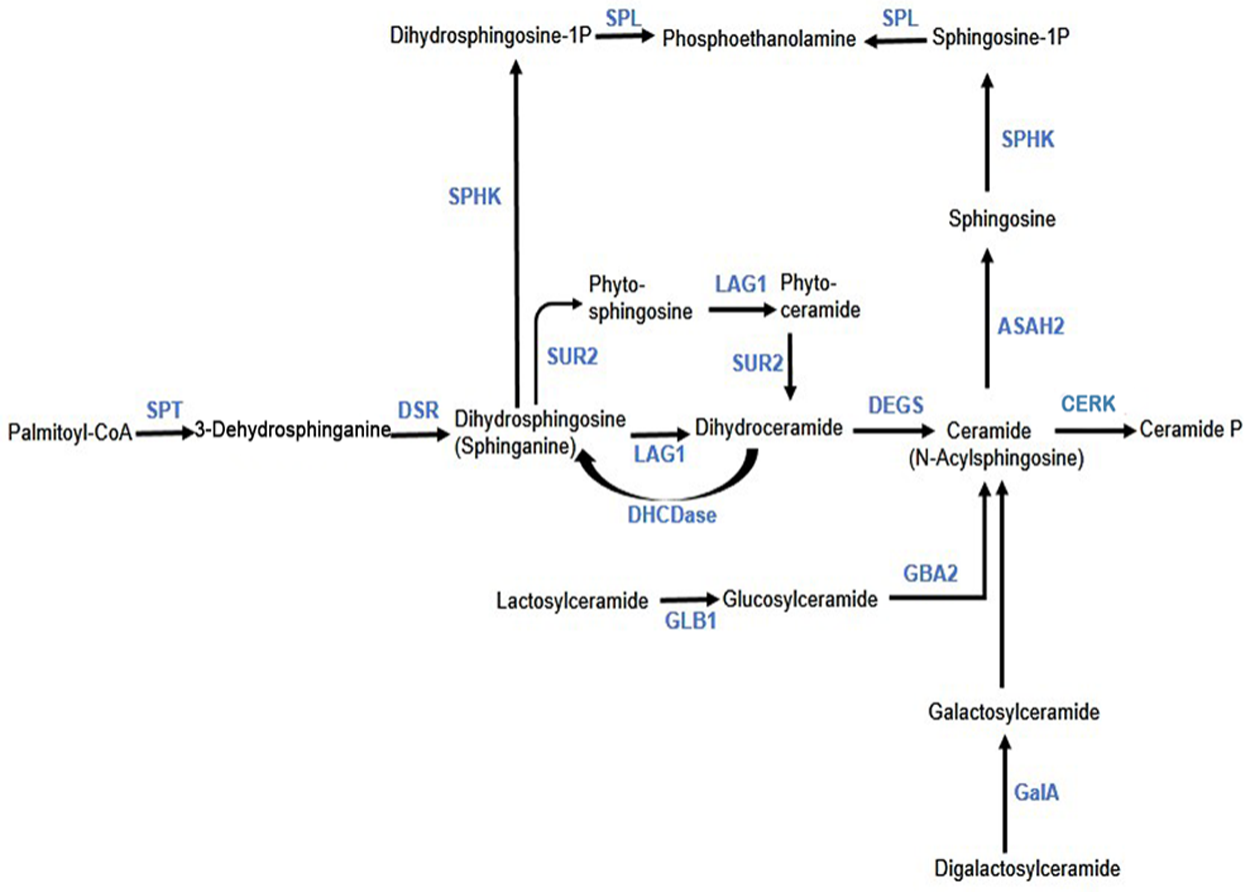
Proposed metabolic pathway of sphingolipids in *A*. *muelleri*. Serine palmitoyltransferase (SPT), 3-Dehydrosphinganine reductase (DSR), sphingosine kinase (SPHK), sphinganine C4-monooxygenase (SUR2), sphinganine-1-phosphate aldolase (SPL), acyl-CoA-dependent ceramide synthase (LAG1), neutral ceramidase (ASAH2), dihydroceramidase (DHCDase), beta-galactosidase (GLB1), sphingolipid delta-4 desaturase (DEGS), non-lysosomal glucosylceramidase (GBA2), alpha-galactosidase (GalA), ceramide kinase (CERK), cellulose synthase-like A (CSLA), cellulose synthase-like D (CSLD), GDPD-pyrophosphorylase (GGP).

In recent years, because of the outbreak of zoonoses such as bovine spongiform encephalopathy (BSE), foot-and-mouth disease (FMD), and avian influenza, the security of animal origin ceramide was of universal concern [22]. *A*. *muelleri* as an important plant source of ceramide has been noticed, and its related biological pathways in *A*. *muelleri* attracted our attention. Assembled transcripts were annotated with corresponding enzyme commission (EC) numbers based on BLASTX alignments in KEGG database. According to the annotation and analysis results of the transcriptome, the possible metabolic pathway of sphingolipids in *A*. *muelleri* wasconstructed (Fig 3), and 13 key enzymes were chosen form sphingolipid metabolic pathways of *A*. *muelleri* (S3 Table).

### Sequence analyses of AmCDase cDNA

**Fig 4:**
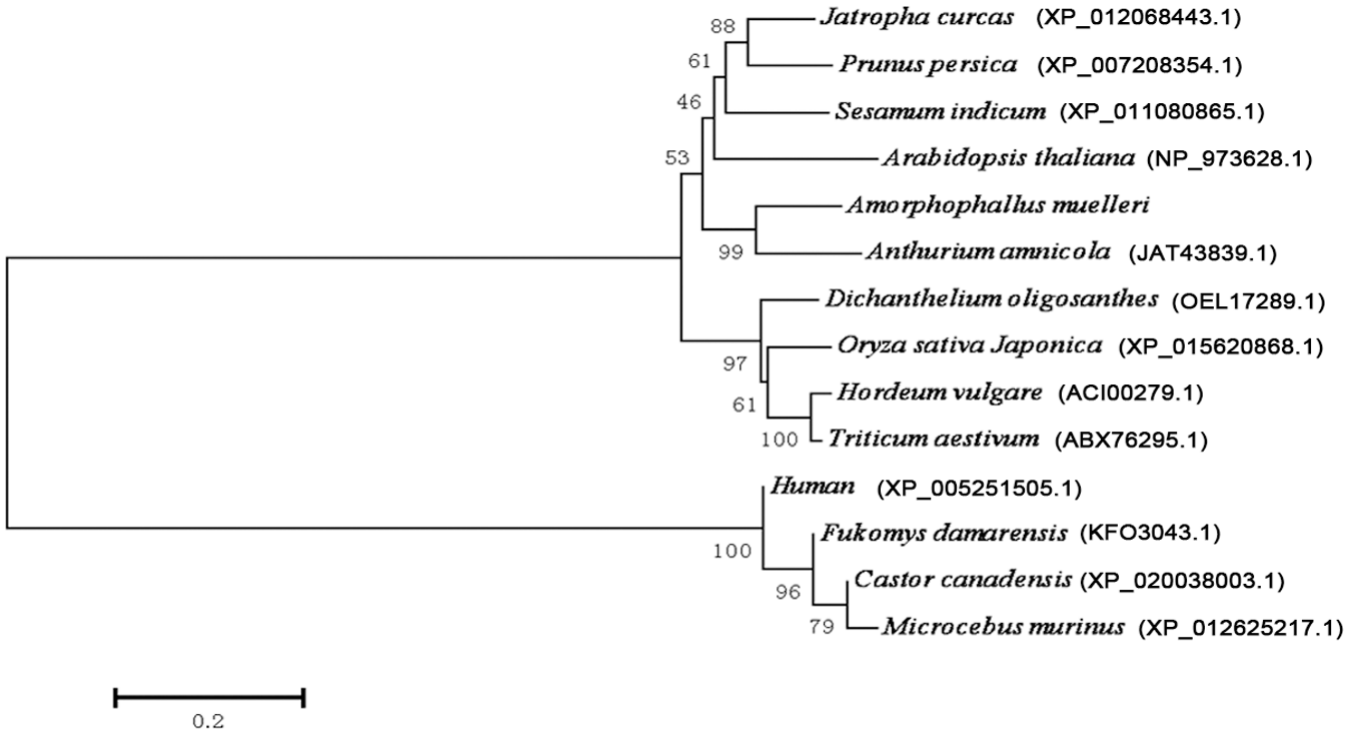
Phylogenetic tree of neutral ceramidases from different species. The MrBayes analyses were performed with a mixed amino acid model. Species names are (*Amorphophallus muelleri; Jatropha curcas; Prunus persica; Sesamum indicum; Arabidopsis thaliana; Anthurium amnicola; Oryza sativa Japonica; Hordeum vulgare; Triticum aestivum; Dicchanthelium oligosanthes; Human; Fukomys damarensis; Microcebus murinus; Castor Canadensis*).

**Fig 5:**
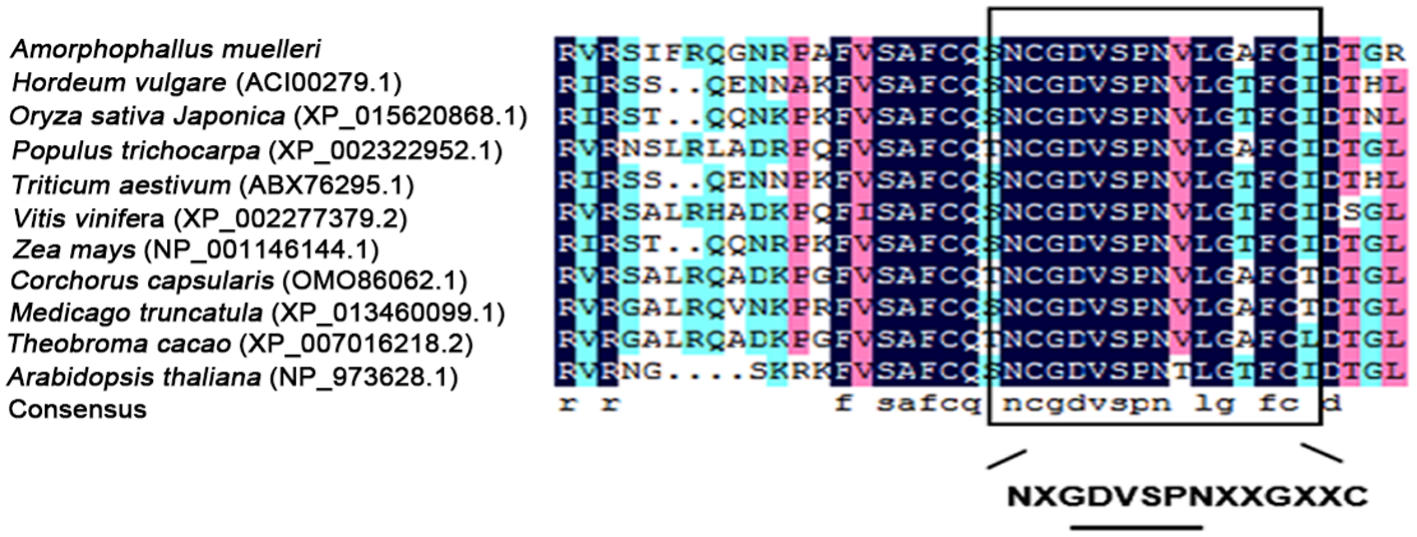
Conserved domain of neutral ceramidases. DNAMAN alignment of the neutral ceramidases from a variety of species showing the highly conserved hexapeptide sequence GDVSPN within the broader conserved amidase domain NXGDVSPNXXGXXC that is crucial for ceramidase activity.

To confirm the identification and properties of this gene, the full-length AmCDase cDNA was obtained. Then, we analysed the nucleotide sequence. The information of the nucleotide was submitted to GenBank (accession no. MF063269). The results showed that the sequence was 2854 bp in length, with an open reading frame 2358 bp encoding a protein of 785 amino acids. The lengths of the 5’-UTR and 3’-UTR are 226 and 270 bp, respectively. The derived amino acid sequence of AmCDase has 83%, 78% and 75% sequence similarity to neutral ceramidase of *Anthurium amnicola*, *Corchorus capsularis*, and *Triticum aestivum*, respectively. All available protein sequences of neutral ceramidases from different species were downloaded from GenBank database (Till 2017.5) and then used to construct the neighbour-joining tree (Fig 4). Phylogenetic analysis of neutral ceramidases from various species show the distance between the plant neutral ceramidase and animal neutral ceramidase. The result also showed that AmCDase was highly related to the neutral ceramidase of many other plant species, such as *Anthurium amnicola*, *Arabidopsis thaliana*, and *Sesamum indicum*. Nevertheless, low correlations could be found between AmCDase and the neutral ceramidase of animals such as humans, *Fukomys damarensis* and *Microcebus murinus*. DNAMAN alignment of the amino acid sequence of AmCDase with ceramidases from various organisms indicated the presence of the highly conserved hexapeptide sequence GDVSPN within the broader conserved amidase domain NXGDVSPNXXCXXG (Fig 5) that is important for ceramidase activity [9, 21]. Meanwhile, the cDNA sequence obtained by RACE was consistent with the related all-unigenes sequences, which showed the ability of *de novo* assembly in discovering transcripts for species without a reference genome.

### Preliminary biochemical characterization of AmCDase

**Fig 6:**
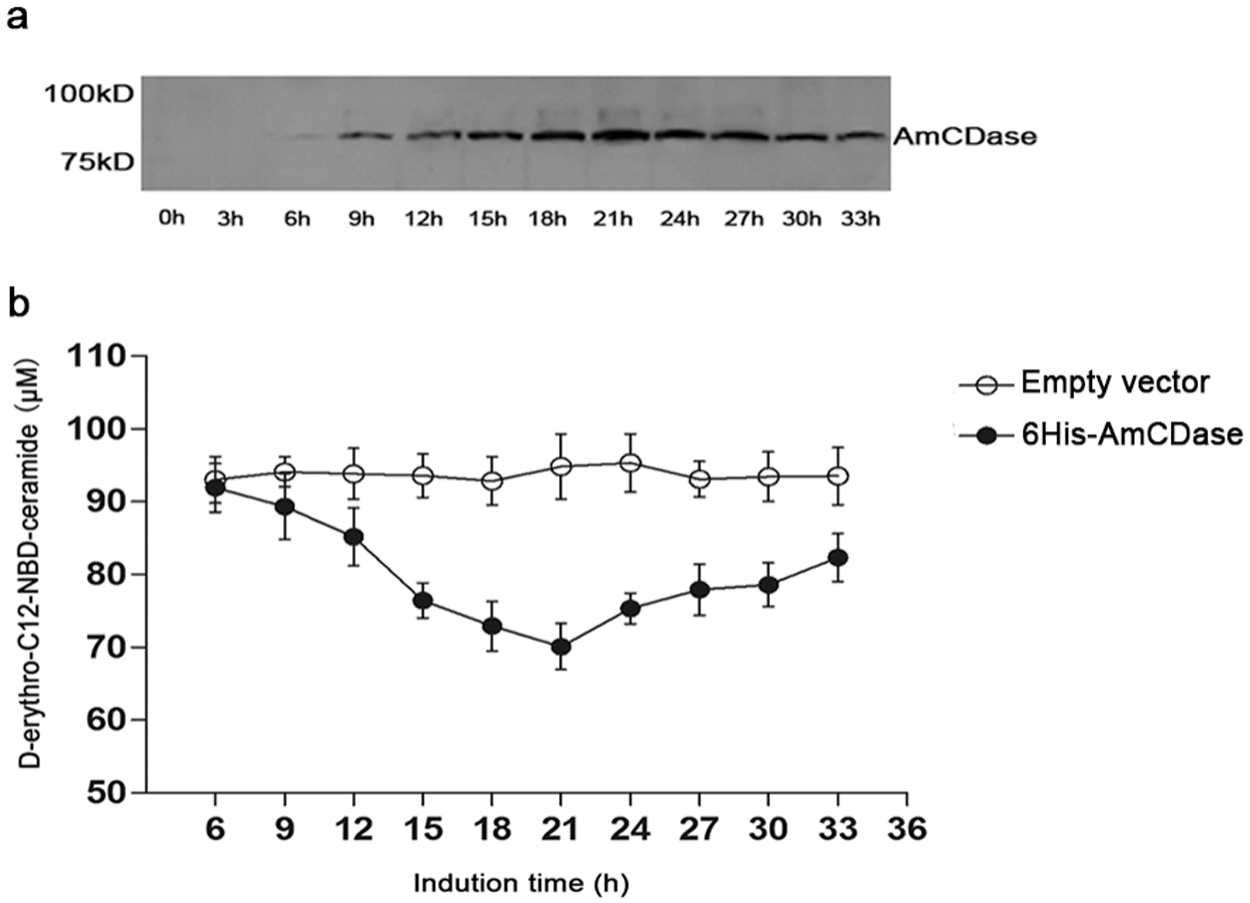
Expression of AmCDase in the yeast double knockout strain Δypc1Δydc1. a) Determination of whole-cell lysates (20 μg protein per lane) by Western blot with anti-His antibody at different time points. b) D-erythro-C12-NBD-ceramide content in different groups after the reaction. Values are the means ± SD of three replicates from each independent experiment.

To explore the biochemical characterization of AmCDase, we subcloned the full-length AmCDase coding sequence and transformed it into the yeast double knockout mutant Δypc1 Δydc1, which lacks yeast ceramidases (the yeast was provided by Professor Fu Xiang-dong from Wuhan University). This is essential for the eukaryotic expression of recombinant AmCDase and could minimize the influence of endogenous yeast ceramidase activity, which is likely to interfere with the following experiments. The level of recombinant AmCDase expression was determined by Western blotting with anti-His antibody (Fig 6a). The results showed that the expression quantity of recombinant AmCDase increased gradually, and the amount of recombinant AmCDase peaked at approximately 21 h, and decreased thereafter. The different induction time yeast with the AmCDase-vector was used in the reactions marked group A, and the yeast with empty vector at the same timing points marked group B. Samples of these two groups reacted with the same amount of substrate (D-erythro-C12-NBD-ceramide) and cell lysates. The enzymatic reaction was continued for the same time under identical conditions, so the difference in the remnant amount of D-erythro-C12-NBD-ceramide could illustrate the degree of reactivity. It showed that D-erythro-C12-NBD-ceramide concentration remained essentially unchanged from the initial concentration in group B after the reaction. However, in group A, the concentration of D-erythro-C12-NBD-ceramide gradually reduced from 6 to 21 h (Fig 6b), and the residual amount of D-erythro-C12-NBD-ceramide was inversely proportional to the addition of AmCDase. The results indicated that the recombinant AmCDase could hydrolyse C12-NBD-Ceramide under certain conditions.

**Fig 7:**
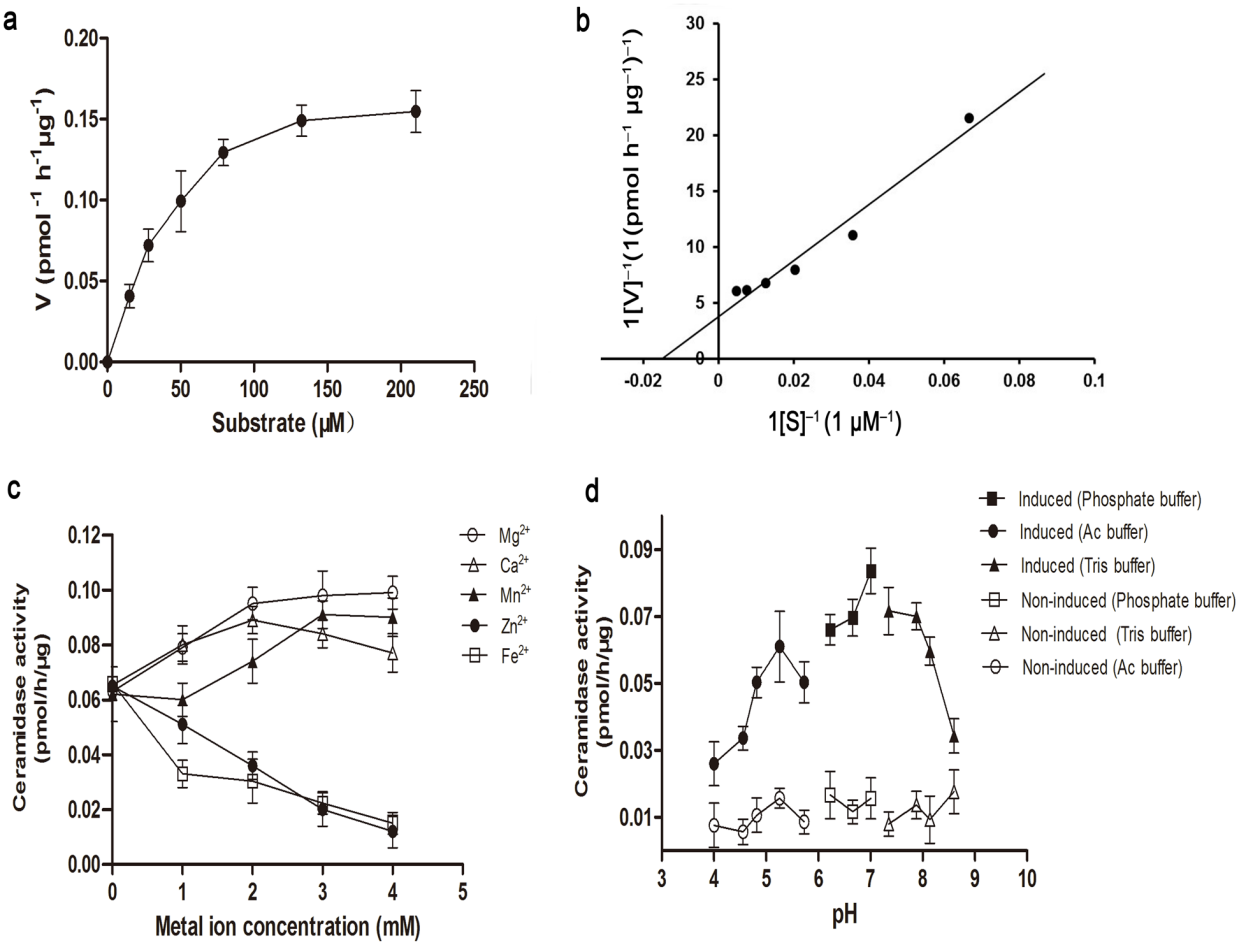
Biochemical characterization of the purified recombinant AmCDase. Hydrolysis of D-erythro-C12-NBD-ceramide was measured by the method described in the “Materials and Methods”. The purified recombinant AmCDase was used for enzyme activity detection, and the reaction continued for 1 h. Values are the means ± SD of three replicates from each independent experiment. (a) Michaelis–Menten represented AmCDase activity by increasing concentrations of D-erythro-C12-NBD-ceramide. (b) Lineweaver-Burk plots for AmCDase. (c) Effects of different cations on AmCDase activity. (d) pH optimum of AmCDase. The buffers were used as described in the experimental procedures.

As seen in Fig 7, the reaction catalysed by purified AmCDase presented classical Michaelis–Menten kinetics (Fig 7a and 7b). The apparent K_m_ and the V_max_ were estimated to be 34.51 μM and 0.20 pmol^-1^h^-1^μg^-1^, respectively, and the K_m_ value was higher than that of the neutral CDases from *Mus musculus* (K_m_ = 22.3μM) [23], but lower than that of *O*. *sativa* (K_m_ = 39.34 μM) [9], *D*. *discoideum* (K_m_ = 38.8 μM) [24] and *Mycobacterium tuberculosis* (K_m_ = 98.7 μM) [25]. AmCDase activity was enhanced by Mg^2+^, Ca^2+^, and Mn^2+^, but inhibited in the presence of Fe^2+^ and Zn^2+^ (Fig 7c). Moreover, AmCDase showed the highest activity between pH 6.5 and 8.0 (Fig 7d). These results demonstrated that the AmCDase was indeed a functional ceramidase, which could hydrolyse the D-erythro-C12-NBD-ceramide.

At present, a great variety of enzymes, which are related to the metabolism of ceramide in plants, have been cloned and characterized. The increasing information makes people more familiar with plant sphingolipids. Numerous studies revealed that the neutral ceramidase widely existed in plants, and it is crucial to ceramide and the whole metabolic pathway of sphingolipids. However, only a few studies about the neutral ceramidase of plants were reported, such as *oryza sativa*, *triticum aestivum*, *arabidopsis thaliana* [9, 26, 27]. In contrast, most of the studies were focused on animals and yeast. In our study, an *A*. *muelleri* neutral ceramidase gene was cloned and its unique biochemical properties were observed. Furthermore, the results showed that the influence of five cations (Mg^2+^, Ca^2+^, Mn^2+^, Zn^2+^ and Fe^2+^) on enzymatic activity in different species was partly consistent. For instance, Mg^2+^, Ca^2+^, and Mn^2+^ could enhance AmCDase activity, and the same goes for the neutral ceramidase in *oryza sativa*. However, the enzymatic activity of neutral ceramidase cannot be activated by Ca^2+^ in *Tribolium castaneum* [25] and *Drosophila* [28]. In addition, Fe^2+^ seems to be a very good inhibitor of the enzymatic activity of neutral ceramidase in *A*. *muelleri*, rice, humans, and *Tribolium castaneum* [22, 25, 28, 29]. Zn^2+^ inhibited the enzymatic activity of AmCDase, which had similar impacts on the neutral ceramidase activity in humans, rats, Drosophila, and Tribolium castaneum. Moreover, the observation indicated that a pH optimum of AmCDase was 6.5–8.0, which was narrower than the pH optimum of the neutral ceramidase of *Dictyostelium discoideum*, but much broader than that of O*ryza sativa* (pH 5.7–6.0), *Drosophila melanogaster* (pH 6.5–7.5), humans (pH 7.5) and the rat kidney (pH 6–7).

There are several studies that have reported the neutral ceramidase of plants, but none of them determined its properties at the protein level by using purified protein. In wheat, the full-length gene of the neutral ceramidase (Ta-CDase) was cloned, and the analyses of the biochemical characterization at the DNA level using Southern blot showed that Ta-CDase was a multi-copy gene and located on wheat chromosome 4D and 5A [25]. Pata et al. tested the biochemical properties of OsCDase by an ingenious method involving heterologous expression in the yeast double knockout mutant without yeast ceramidases, but the purified enzyme was not performed in further research [9]. The detailed analysis based on the purified enzyme was more accurate and persuasive. In our study, we improved the method of Pata et al. by using the purified enzyme to determine the characterization of AmCDase and demonstrated that AmCDase was a real neutral ceramidase. As early as nine years ago, the experts of this field proposed that deeper understanding of the roles that sphingolipids play in plant physiological function would come from a better understanding at the transcriptomic level [11]. This study was exactly based on the data of transcriptome analysis. Furthermore, with mounting transcriptome data from various species, it was expected that valuable roles of plant sphingolipids would emerge as increasing plant sphingolipidomes are characterized accurately and quickly.

## Conclusions

In this study, we found the possible sphingolipid metabolic pathway and 13 enzymes involved in it. Then, the key enzyme-neutral ceramidase (AmCDase) was cloned and its biochemical characterization was analysed. The results showed that the reaction catalysed by AmCDase presented classical Michaelis–Menten kinetics, and the influences of cations on the enzyme activity of the neutral ceramidase varied with the difference in species. In addition, the transcriptome analyses can provide accurate and specific data for the gene clone, which revealed the powerful ability of high-throughput sequencing in identifying genes from non-model organisms. Our research demonstrated that *de novo* transcriptome assembly can be considerable for certain purposes, such as transcriptome data mining, gene cloning and so on. In addition, sequencing data can provide basic information for further research about neutral ceramidases and a comprehensive sequence resource for *A*. *muelleri* study, such as gene expression, genomics, and functional genomics in *Amorphophallus*.

## Supporting information

S1 Fig Statistics of contig assembly qualities. All sizes of the Unigenes were calculated.
(TIF)

S2 Fig KOG function classification of the Unigenes. (TIF)

S1 Table Summary of the transcriptome of *A*. *muelleri*. (DOCX)

S2 Table BLAST analysis results against important public databases. (DOCX)

S3 Table Candidate genes related to the ceramide metabolism pathway in *A*. *muelleri*. (DOCX)
