# Supplementary figures and images for: Gene cloning of a neutral ceramidase from the sphingolipid metabolic pathway based on transcriptome analysis of Amorphophallus muelleri
Source: PLoS One. 2018 Mar 28;13(3):e0194863. doi: 10.1371/journal.pone.0194863 (PMC5874051; doi:10.1371/journal.pone.0194863)

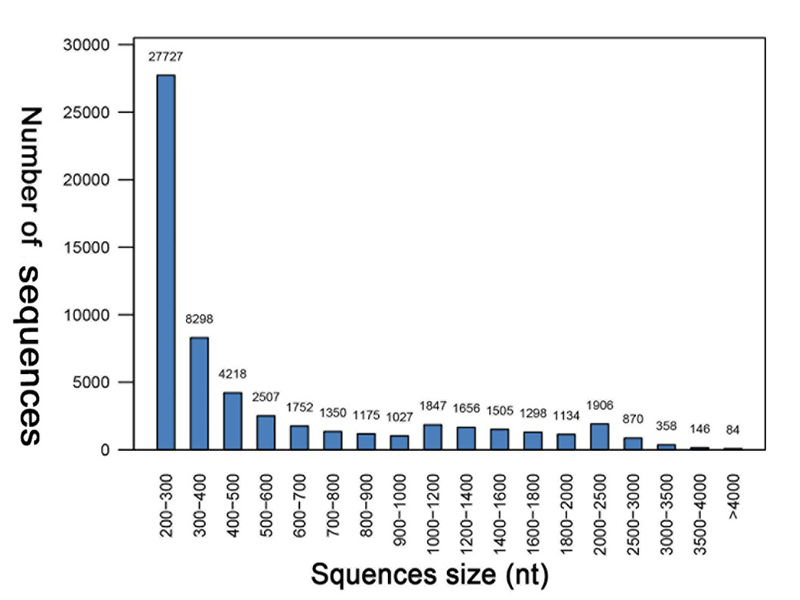

Supplement: S1 Fig — All sizes of the Unigenes were calculated. (TIF) [file pone.0194863.s001.tif]

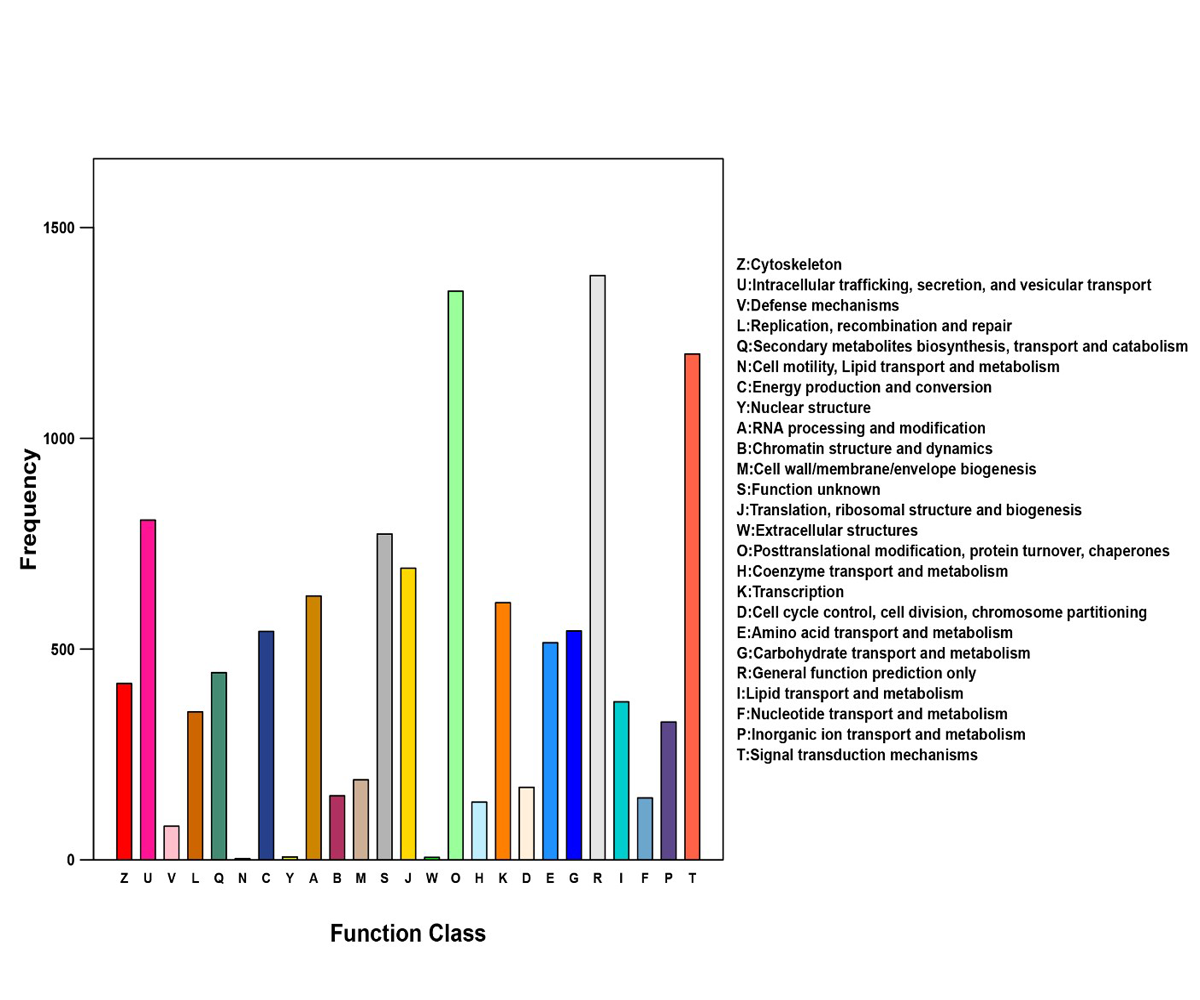

Supplement: S2 Fig — (TIF) [file pone.0194863.s002.tif]
