# Supplementary material for: Gene cloning of a neutral ceramidase from the sphingolipid metabolic pathway based on transcriptome analysis of Amorphophallus muelleri
Source: PLoS One. 2018 Mar 28;13(3):e0194863. doi: 10.1371/journal.pone.0194863 (PMC5874051; doi:10.1371/journal.pone.0194863)
Supplement: S1 Table — (DOCX) [file pone.0194863.s003.docx]

**S1 Table. Summary of the transcriptome of A.muelleri**

| **Sample** | **Total reads** | **Total clean Nucleotides (nt)** | **Total Q20 percentage** | **GC percentage** | **Total number of transcripts** | **Average length of transcripts (nt)** | **N50 of transcripts** | **Total number of unigenes** | **Average length of unigenesn (nt)** | **N50 of unigenes** |
| --- | --- | --- | --- | --- | --- | --- | --- | --- | --- | --- |
| *A.muelleri* | 15867314 | 15132167 | 97.8 | 50.3 | 85775 | 795 | 1391 | 58858 | 618 | 1127 |
