# Supplementary material for: Gene cloning of a neutral ceramidase from the sphingolipid metabolic pathway based on transcriptome analysis of Amorphophallus muelleri
Source: PLoS One. 2018 Mar 28;13(3):e0194863. doi: 10.1371/journal.pone.0194863 (PMC5874051; doi:10.1371/journal.pone.0194863)
Supplement: S2 Table — (DOCX) [file pone.0194863.s004.docx]

**S2 Table. BLAST analysis results against important public databases**

| **Sample** | **Number of All unigenes** | **NR(%)** | **Swiss-Prot(%)** | **KEGG(%)** | **KOG(%)** |
| --- | --- | --- | --- | --- | --- |
| *A.muelleri* | 58851 | 21675（36.83%） | 15017（25.51%） | 21239（36.09%） | 11848（20.13%） |
