# Supplementary material for: Gene cloning of a neutral ceramidase from the sphingolipid metabolic pathway based on transcriptome analysis of Amorphophallus muelleri
Source: PLoS One. 2018 Mar 28;13(3):e0194863. doi: 10.1371/journal.pone.0194863 (PMC5874051; doi:10.1371/journal.pone.0194863)
Supplement: S3 Table — (DOCX) [file pone.0194863.s005.docx]

**S3 Table. Candidate genes related to ceramide metabolism pathway in A. muelleri**

| Enzyme | Number of EC | Symbol |
| --- | --- | --- |
| Serine palmitoyltranferase | 2.3.1.50 | SPT |
| 3-Dehydrosphinganine reductase | 1.1.1.102 | DSR |
| Sphingosine kinase | 2.7.1.91 | SPHK |
| Sphinganine C4-monooxygenase | 1.14.13.169 | SUR2 |
| Sphinganine-1-phosphate aldolase | 4.1.2.27 | SPL |
| Acyl-CoA-dependent ceramide synthase | 2.3.1.24 | LAG1 |
| Neutral ceramidase | 3.5.1.23 | ASAH2 |
| Dihydroceramidase | 3.5.1.- | DHCDase |
| Beta-galactosidase | 3.2.1.23 | GLB1 |
| Sphingolipid delta-4 desaturase | 1.14.-.- | DEGS |
| Non-lysosomal glucosylceramidase | 3.2.1.45 | GBA2 |
| Alpha-galactosidase | 3.2.1.22 | GalA |
| Ceramide kinase | 2.7.1.138 | CERK |
